# Editorial: Be positive about the negative in pharmacology: Neuropharmacology 2022

**DOI:** 10.3389/fphar.2023.1256508

**Published:** 2023-10-09

**Authors:** Adrian Preda, Rene Oliveira Beleboni

**Affiliations:** ^1^ Psychiatry and Human Behavior, University of California, Irvine, Irvine, CA, United States; ^2^ Biotechnology Department/School of Medicine, University of Ribeirão Preto, Ribeirão Preto-SP, Brazil

**Keywords:** publication bias, negative findings, results reproducibility, scientific integrity, research transparency

Our “*Be Positive about Negative Results in Pharmacology*” Research Topic seeks to illuminate data that often remain unpublished. Unpublished results are typically divided into two categories.

1. Uninterpretable results, stemming from inadequately designed experiments, flawed data Research Topic, or incorrect analysis.
2. Solid negative results, derived from robustly designed, unbiased experiments with accurate analysis.

Uninterpretable results do not warrant publication, but solid results, regardless of their nature, should be published (Bespalov et al., 2019). However, the reluctance of many studies to publish negative results, even when robustly designed, is common (Matosin et al., 2014).

This hesitancy is often due to the perceived difficulty in distinguishing a true negative result from an uninterpretable one. Unknown confounding factors could compromise an apparently solid negative result, leading to potential misinterpretation.

This reluctance to publish solid negative results, while understandable, contributes to a “positive publication bias” (Fanelli, 2010). Such bias favors positive results, as they are often seen as more significant in advancing knowledge. This inclination, however, leads to several harmful consequences.

• Incomplete scientific knowledge: only publishing positive results creates a skewed understanding of the efficacy and safety of pharmacological interventions.
• Wasteful use of resources: when negative results remain unpublished, the scientific community may repeat unsuccessful studies, wasting time, funding, and other resources.
• Patient safety and ethical concerns: concealing negative studies can affect healthcare providers’ treatment decisions, leading to patients receiving unsafe or ineffective treatments.
• Biased meta-analyses: a surplus of positive results can distort the scientific record, influencing meta-analyses and systematic reviews.

To address these Research Topic, we present our Research Topic “*Be Positive about Negative Results in Pharmacology*.” Here are the highlights of our negative studies Research Topic.

1. LMH001 does not appear to significantly impact NOX_2_ pharmacology, and thus is unlikely to be an effective intervention in mitigating oxidative stress and inflammation.

Originally believed to impact NOX_2_ pharmacology by reducing oxidative stress and inflammation, LMH001 was found to be chemically unstable in the study by Zang et al. This lack of stability, combined with weak inhibition of NOX_2_, raised doubts about LMH001s effectiveness and mechanism.

2. Functional MRI static and dynamic functional connectivity predict ECT-antidepressant response but not ECT-associated cognitive changes.

Functional MRI static and dynamic functional connectivity effectively predict ECT-antidepressant response but fail to predict ECT-associated cognitive changes, according to a study by Fu et al. ECT is a potent treatment for treatment-resistant depressive episodes but has notable cognitive side effects. The authors’ exploration of functional connectivity, via both static and dynamic perspectives, seeks to predict who will respond to ECT and who might risk cognitive impairment.

By analyzing an ECT dataset using a fully automated independent component analysis framework, the authors extracted static and dynamic FNC data and developed predictive models. Their results reveal that changes in both sFNC and dFNC predict antidepressant outcomes and memory changes. Dynamic functional connectivity, however, did not significantly improve memory change prediction. These findings affirm the value of integrating dynamic functional connectivity analysis to better comprehend ECT’s mechanisms and outcomes.

3. Pharmacological modulation of circadian rhythms for the prevention of IBD pathogenesis may not be warranted.

Chen et al. explored the relationship between sleep changes and IBD. Using a two-sample Mendelian randomization study, the authors concluded that various sleep traits do not causally affect IBD. This suggests that the pharmacological modulation of circadian rhythms may not prevent IBD pathogenesis. Future research should investigate other potential factors contributing to IBD development and progression, highlighting the complexity of IBD etiology.

4. Gabapentin and pregabalin correlate with an increased risk for dementia.

The study by Huang et al. investigated the link between gabapentin or pregabalin use and dementia risk. The authors found a significant correlation, with exposed patients facing a 45% higher risk compared to their non-exposure counterparts. This association prompts the need for careful evaluation of these medications’ therapeutic benefits against their associated risks. Further research is necessary to explore the underlying mechanisms of this observed association and to possibly shed light on dementia’s development and prevention.

## Conclusion

The “*Be Positive about the Negative in Pharmacology*” Frontiers Research Topic presents articles that emphasize the importance of negative results from a variety of perspectives. Our range of Research Topic and methods is broad: from molecular biology to brain imaging to circadian rhythms to epidemiological studies. The major findings from the reports in this Research Topic are.

1. LMH001 does not appear to have a significant impact on NOX_2_ pharmacology, and thus is unlikely to be an effective intervention in mitigating oxidative stress and inflammation Zang et al.
2. Functional MRI static and dynamic functional connectivity predict ECT-antidepressant response but not ECT-associated cognitive changes Fu et al.
3. The modulation of circadian rhythms for the prevention of IBD pathogenesis may not be warranted Chen et al.
4. Gabapentin and pregabalin are correlated with an increased risk for dementia Huang et al.

The articles in this Research Topic illustrate the multitude of perspectives that necessarily complete one another and improve our understanding of the neurobiological complexity underlying current neuro-psychopharmacological interventions. Each article also presents a unique negative findings perspective, which we find informative in bettering our understanding of the addressed Research Topic, as compared to previously reported positive findings.
